# Circulatory levels of alarmins in patients with non-segmental vitiligo: Potential biomarkers for disease diagnosis and activity/severity assessment

**DOI:** 10.3389/fimmu.2022.1069196

**Published:** 2022-12-07

**Authors:** Kaiqiao He, Wei Wu, Xinju Wang, Wei Dai, Sijia Wang, Chunying Li, Shuli Li

**Affiliations:** ^1^ Department of Dermatology, Xijing Hospital, Fourth Military Medical University, Xi’an, China; ^2^ Department of Dermatology and Venereology, Nanfang Hospital, Southern Medical University, Guangzhou, China

**Keywords:** vitiligo, alarmins, biomarkers, diagnose, disease activity, disease severity

## Abstract

**Background:**

Non-segmental vitiligo (NSV) is an autoimmune skin disorder that is difficult to determine disease activity/severity and thus to treat. Alarmins have emerged as promising biomarkers in various diseases, so further confirmation of their potential roles in NSV would be of considerable value. With the present work, we aimed to determine the serum levels of alarmins in patients with NSV, correlate these alarmins with disease activity and severity, and analyze the predictive value of the combination of these markers.

**Methods:**

104 NSV patients and 56 healthy controls were enrolled at the Xijing Hospital of Fourth Military Medical University between September 1, 2018, and June 30, 2019. The serum levels of alarmins (including IL-33, IL-1α, S100A9, S100A12, S100B, and HMGB1) were measured with enzyme-linked immunosorbent assays. The predictive performance of these biomarkers was evaluated with the area under the receiver operating characteristic curve (AUC), sensitivity, specificity, and other representative statistics.

**Results:**

A total of 104 patients with NSV (mean [SD] age, 34.2 [13.0] years; 62 [59.6%] male) and 56 healthy controls (mean [SD] age, 34.8 [13.5] years; 34 [60.7%] male) were enrolled. For vitiligo diagnosis, S100B had the highest sensitivity (92.31%), whereas HMGB1 had the highest specificity (85.71%); the combination of IL-1α, S100B, S100A9, and HMGB1 increased the AUC value to 0.925, with a sensitivity of 87.50% and a specificity of 85.71%. Multivariate logistic regression analysis showed S100B (OR, 1.019; 95% CI, 1.002-1.038; *P* =0.03), S100A9 (OR, 1.002; 95% CI, 1.001-1.003; *P*<0.001), and HMGB1 (OR, 1.915; 95% CI, 1.186-3.091; *P* =0.008) were significantly associated with vitiligo activity. S100A9 had the highest accuracy in discriminating patients at the active stage from the stable stage, with an AUC value of 0.827. The combination of these alarmins had an AUC value of 0.860 to assess disease activity, with a sensitivity of 90.00% and a specificity of 72.97%. Furthermore, S100B (r=0.61, *P <*0.001), S100A9 (r=0.33, *P <*0.001), and HMGB1 (r = 0.51, *P <*0.001) levels were positively correlated with the affected body surface area (BSA) in NSV patients.

**Conclusions:**

Serum S100B, S100A9, and HMGB1 might be biomarkers for diagnosing and assessing the activity/severity of NSV, either used alone or in combination.

## Introduction

Vitiligo is a common depigmented skin disease with a worldwide prevalence of 0.5%-2% (1, 2). It can be classified into two major forms: non-segmental vitiligo (NSV, also known as vitiligo) and segmental vitiligo (SV). SV only accounts for 5% to 16% of all vitiligo cases and is characterized by sudden onset with rapid stabilization, whereas NSV is the most common form with unpredictable course, which brings patients significant psychosocial and economic detriment (3, 4). The burden imposed by vitiligo is further exacerbated by a lack of objective methods to gauge the activity and severity of the disease and subsequently a belated or imprecise management. Clinically, the diagnosis and assessment of vitiligo are highly dependent on the physician’s subjective judgment, which mainly rely on cutaneous symptoms like the trichrome sign, confetti-like depigmentation, and the Koebner phenomenon (5). However, cutaneous symptoms cannot be recognized in all patients and are always imperceptible at the onset, making it imperative to establish biomarkers that may be detected early and reliably assess the disease status. Recent studies have discovered several serum biomarkers for vitiligo, including sCD25 and sCD27 (6). The use of such serum markers for vitiligo assessment would help guide better management.

Alarmins are endogenous, constitutively expressed, chemotactic, and immune-activating proteins/peptides. They have been thought to be initiators of both innate and adaptive immunity, as well as influencing the types of adaptive immune responses (7, 8). In response to cell injury or death, alarmins are released to galvanize immune cells both in host defense and disease (9). Increasing studies have discovered that alarmins could also serve as biomarkers for the diagnosis, prognosis, and treatment response in several autoimmune diseases, including rheumatoid arthritis, systemic lupus erythematosus, and psoriatic arthritis (10–12). Among these alarmins, HMGB1 and S100B have been reported to be involved in the pathogenesis of vitiligo and could even serve as potential therapeutic targets for vitiligo (13, 14). The serum concentrations of IL-1α and IL-33 were reported higher in vitiligo patients than that in healthy subjects (15, 16). S100A9 and S100A12, the two alarmins are prone to rise after oxidative stress which is an important inducer for vitiligo initiation, and then involved in the aberrant immune response (17, 18). Therefore, in this study we would like to detect the serum expression of these above alarmins (HMGB1, S100B, IL-1α, IL-33, S100A9, and S100A12) in NSV patients and healthy controls, to further investigate their clinical significance in the diagnosis and assessment of disease activity/severity in vitiligo.

## Materials and methods

### Participants

In this cross-sectional study, one hundred and four patients with NSV were enrolled at the Department of Dermatology, Xijing Hospital of Fourth Military Medical University between September 1, 2018, and June 30, 2019. Exclusion criteria included a diagnosis of other autoimmune diseases aside from vitiligo, and with any immunosuppressive or narrow band Ultra Violet B (NB-UVB) therapy within 6 weeks before enrollment to the study. In addition, fifty-six age and sex-matched healthy controls were recruited from the physical examination. None of the healthy subjects had a personal history of autoimmune disorder or any previous treatment influencing inflammatory response. Written informed consent was obtained from each patient and healthy control before the study. The study was designed and executed according to the principles of the Declaration of Helsinki and approved by the ethics committee of Xijing Hospital of Fourth Military Medical University.

### Assessment of vitiligo disease activity

Disease activity was assessed by three dermatologists according to “Diagnosis and treatment of vitiligo: an expert consensus statement (2018)” published in Chinese Journal of Dermatology and classified into three categories: stable, mild-moderate active, and very active (19).

Stable vitiligo may be determined if at least two items were identified: VIDA score ≤ 0; clinical feature of lesions with clear edges or signs of repigmentation; no Koebner phenomenon within 1 year; white lesion with sharply clear borders, smaller than or equal to the visual area under Wood’s light.

Any one of the following four characteristics might indicate active vitiligo: VIDA score 1 to 4; clinical features, including poorly defined borders and inflammatory signs (such as trichromatic vitiligo, confetti-like depigmentation, and hypopigmentation); Koebner phenomenon in the past 1 year; In the Wood’s light examination, poorly demarcated borders associated with hypomelanotic edging or larger hypochromia area than the visual area. Among active vitiligo, mild-moderate active vitiligo was scored with VIDA score 1 to 3, and very active vitiligo in patients with VIDA score equal to 4 or obvious clinical markers.

### Assessment of vitiligo disease severity

Disease severity was assessed based on the affected body surface area (BSA) and evaluated by three experts independently. One hand unit is about 1% of the BSA; one finger unit accounts for 0.1% of the BSA; one fingertip unit is equal to 0.03% of the BSA approximately. The final BSA values were averaged among the experts.

### Blood samples collection and laboratory measurements

The samples of peripheral blood from patients with NSV and healthy controls were collected, and the serum was isolated by centrifugation for 5 minutes at 1000×g. After that, samples were stored under -80°C until analysis. Repeated thawing and freezing were avoided. Commercial enzyme-linked immunosorbent assays (ELISA) were used to measure the serum level of IL-33, IL-1α, S100A12 (Beijing 4A Biotech Co., Ltd), S100A9, S100B (R&D Systems), and HMGB1 (IBL, Japan).

### Statistical analysis

Statistical analysis was conducted using SPSS, version 26 (IBM Corporation), and GraphPad Prism, version 9 (GraphPad Software). Continuous variables were expressed as mean (SD) or median (interquartile range) and discrete variables were expressed as percentage distributions. Two independent samples t-test and chi-square test were used to compare the differences between NSV and healthy controls. Mann-Whitney U test and Kruskal-Wallis H test were used to compare the differences between two or more than two groups. The receiver operating characteristic curve was used to investigate the sensitivity and specificity of alarmins as biomarkers. Adjustment of possible confounding factors was performed using multivariate logistic regression. For correlation analysis, Pearson correlation analyses were performed. In all cases, *P*<0.05 was considered statistically significant.

## Results

### Clinical characteristics of the participants

A total of 104 patients with NSV (mean [SD] age, 34.2 [13.0] years; 62 [59.6%] male) and 56 healthy controls (mean [SD] age, 34.8 [13.5] years; 34 [60.7%] male) were included in the study. Subjects were well-matched for age and sex. In addition, 30 of 104 patients (28.8%) had stable vitiligo, 37 of 104 patients (35.6%) had mild-moderate active vitiligo, and the 37 remaining patients (35.6%) had very active vitiligo (**Table 1**).

**Table 1:** Baseline demographics and clinical characteristics.

Characteristics	Patients with NSV (n=104)	Controls (n=56)	*P*-value
Age (years), mean (SD)	34.2 (13.0)	34.8 (13.5)	0.76^a^
Sex, n (%)			0.89^b^
Female	42 (40.4%)	22 (39.3)	NA
Male	62 (59.6%)	34 (60.7)	NA
Age at disease onset (years), mean (SD)	24.5 (13.0)	NA	NA
Disease duration (months), median (IQR)	72 (36-165)	NA	NA
Activity, n (%)		NA	NA
Stable	30 (28.8%)	NA	NA
Mild-moderate active	37 (35.6%)	NA	NA
Very active	37 (35.6%)	NA	NA
Affected BSA (%), median (IQR)	6 (2-25.9)	NA	NA
Serum alarmins, median (IQR)	NA	NA	NA
IL-33, pg/ml	19.0 (16.0-25.7)	19.0 (15.7-27.3)	0.818^c^
IL-1α, pg/ml	6.7 (5.5-9.7)	4.9 (4.3-5.6)	**<0.001^c^**
S100A12, ng/ml	9.4 (8.5-10.3)	8.8 (7.4-10.4)	0.109^c^
S100B, pg/ml	31.3 (20.8-47.2)	19.9 (7.5-30.9)	**<0.001^c^**
S100A9, ng/ml	1171.4 (790.1-1690.3)	623.7 (396.8-1169.7)	**<0.001^c^**
HMGB1, ng/ml	1.5 (0.9-2.0)	0.8 (0.5-1.1)	**<0.001^c^**

BSA, body surface area; SD, standard deviation; IQR, interquartile range; NA, not applicable.

^a^P value was calculated by Independent samples t-test.

^b^P value was calculated by Chi-square test.

^c^P value was calculated by Mann-Whitney U test p-value <0.05 was represented in bold.

### Serum levels of IL-1α, S100B, S100A9, and HMGB1 are increased in patients with NSV

**Figure 1:**
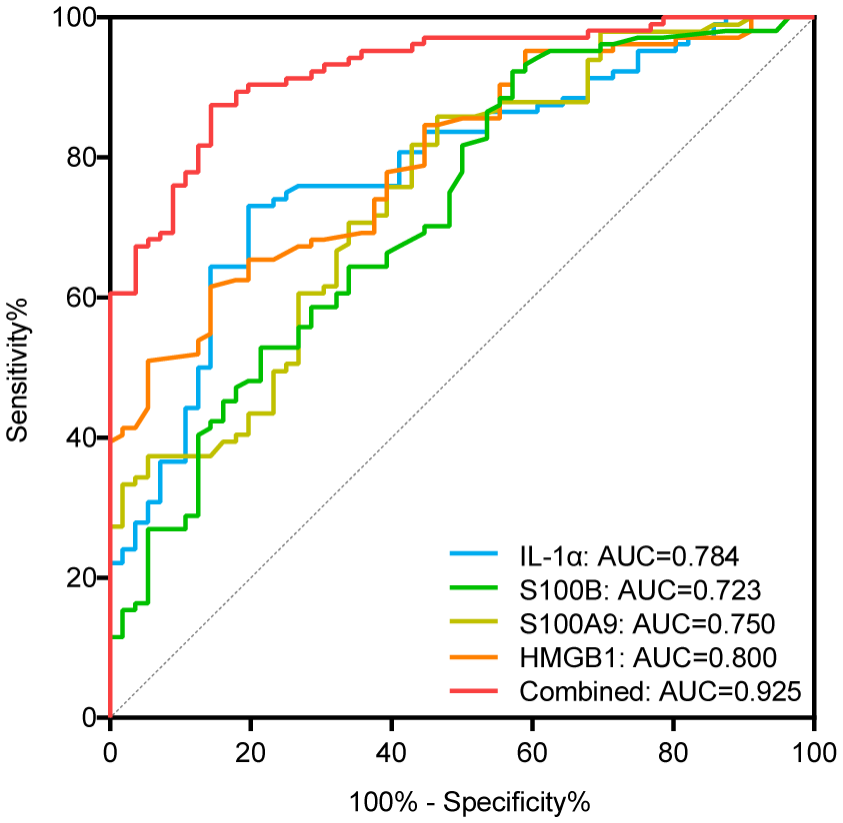
ROC curves of IL-1α, S100B, S100A9, and HMGB1 for diagnosing vitiligo. AUC indicates the area under the receiver operating characteristic curve. The blue, green, yellow, orange curve represents the ROC curve of IL-α, S100B, S100A9, and HMGB1, respectively; the red curve represents the ROC curve of the combination of IL-1α, S100B, S100A9, and HMGB1.

Regarding the expression of alarmins, serum levels of IL-1α, S100B, S100A9, and HMGB1 were significantly elevated in patients with NSV compared with healthy controls, whereas the expression of IL-33 and S100A12 showed no change (**Table 1**). The ROC curve analysis for identifying disease diagnostic value was significant for IL-1α, S100B, S100A9, and HMGB1, with AUC values of 0.784, 0.723, 0.750, and 0.800, respectively. Among these alarmins, S100B had the highest sensitivity of 92.31% and HMGB1 had the highest specificity of 85.71%. The diagnosis accuracy for each combination of IL-1α, S100B, S100A9, and HMGB1 was also carried out. When the four biomarkers were combined, the AUC value was 0.925, with a sensitivity of 87.50% and a specificity of 85.71%, which was superior to using any biomarker alone or any kind of combination (**Table 2**, **Figure 1**).

**Table 2:** Diagnostic values of biomarkers in patients with NSV.

Alarmins	Cut-off value	AUC (95%CI)	Sensitivity, %	Specificity, %	PPV, %	NPV, %
IL-1α, pg/ml	5.647	0.784 (0.711,0.857)	73.08	80.36	87.36	61.65
S100B, pg/ml	15.32	0.723 (0.640,0.806)	92.31	42.86	75.0	75.01
S100A9, ng/ml	628.544	0.750 (0.673,0.828)	85.58	53.57	77.39	66.67
HMGB1, ng/ml	1.227	0.800 (0.733,0.867)	61.54	85.71	88.89	54.55
IL-1α+S100B		0.823 (0.758,0.888)	75.96	76.77	85.86	63.23
IL-1α+S100A9		0.848 (0.787,0.908)	88.46	67.86	83.64	76.00
IL-1α+HMGB1		0.890 (0.841,0.939)	78.85	85.71	91.11	68.57
S100B+S100A9		0.805 (0.739,0.871)	59.62	94.64	95.38	55.79
S100B+HMGB1		0.848 (0.789,0.907)	62.50	92.86	94.21	57.14
S100A9+HMGB1		0.863 (0.808,0.918)	69.23	89.29	92.31	60.98
IL-1α+S100B+S100A9		0.864 (0.808,0.919)	71.15	85.71	90.24	61.53
IL-1α+S100B+HMGB1		0.900 (0.854,0.947)	84.62	80.36	88.89	73.78
S100B+S100A9+HMGB1		0.884 (0.834,0.934)	77.88	87.50	92.05	68.05
all combined		0.925 (0.886,0.965)	87.50	85.71	91.92	78.69

AUC, area under the curve; PPV, positive predictive value; NPV, negative predictive value.

The diagnostic cut-off value corresponds to the maximum point of the Youden index.

The all combined represents the combination of IL-1α, S100B, S100A9, and HMGB1.

### Serum levels of S100B, S100A9, and HMGB1 are increased in active patients with NSV

**Figure 2:**
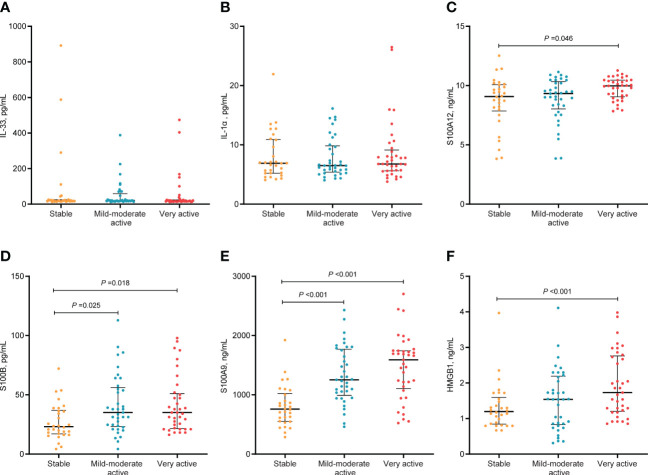
Serum levels of alarmins in different activity stage in patients with NSV. The median and IQR were represented by the middle black line, and the lower and upper black lines, respectively. Serum levels of IL-33 **(A)**, IL-α **(B)**, S100A12 **(C)**, S100B **(D)**, S100A9 **(E)**, HMGB1 **(F)** in patients with NSV at stable, mild-moderate active, and very active stage.

In terms of disease activity evaluated by physicians, we found that the serum levels of S100A12, S100B, S100A9, and HMGB1 in patients with very active vitiligo were significantly higher than those in patients in stable phase (S100A12, *P*=0.046; S100B, *P*=0.018; S100A9, *P*<0.001; HMGB1, *P*<0.001). In addition, patients in mild-moderate phase had higher levels of S100B and S100A9 than those in stable phase (S100B, *P* =0.025; S100A9, *P*<0.001). However, no obvious difference was observed between patients in mild-moderate active phase and very active phase (**Figure 2**). To further eliminate the impact of confounding factors on our results, including age, age at disease onset, and disease duration, we conducted multivariate logistic regression. Our results showed that the serum levels of S100B (OR, 1.019; 95% CI, 1.002-1.038; *P* =0.03), S100A9 (OR, 1.002; 95% CI, 1.001-1.003; *P*<0.001), and HMGB1 (OR, 1.915; 95% CI, 1.186-3.091; *P* =0.008) were significantly associated with disease activity (**Table 3**).

**Table 3:** Multivariate models investigating the association of S100A12, S100B, S100A9, HMGB1, and disease activity.

Independent variable	Coefficient	Wald test	OR (95% CI)	*P*-value
Model 1				
Age	1.414	2.065	4.111 (0.598, 28.272)	0.15
Disease duration	-0.112	1.884	0.894 (0.763, 1.049)	0.17
Age at disease onset	-1.393	2.003	0.248 (0.036, 1.710)	0.16
S100A12	0.214	3.458	1.238 (0.989, 1.550)	0.06
Model 2				
Age	1.577	2.592	4.840 (0.710, 33.007)	0.11
Disease duration	-0.124	2.364	0.883 (0.753, 1.035)	0.12
Age at disease onset	-1.556	2.521	0.211 (0.031, 1.440)	0.11
S100B	0.019	4.652	1.019 (1.002, 1.038)	**0.03**
Model 3				
Age	1.640	2.441	5.156 (0.659, 40.347)	0.12
Disease duration	-0.130	2.234	0.878 (0.741, 1.041)	0.14
Age at disease onset	-1.620	2.377	0.198 (0.025,1.552)	0.12
S100A9	0.002	21.816	1.002 (1.001,1.003)	**<0.001**
Model 4				
Age	1.280	1.721	3.598 (0.531, 24.364)	0.19
Disease duration	-0.100	1.535	0.905 (0.773, 1.060)	0.22
Age at disease onset	-1.264	1.674	0.283 (0.042, 1.917)	0.20
HMGB1	0.650	7.063	1.915 (1.186, 3.091)	**0.008**

OR, odds ratio.

p-value <0.05 was represented in bold.

**Figure 3:**
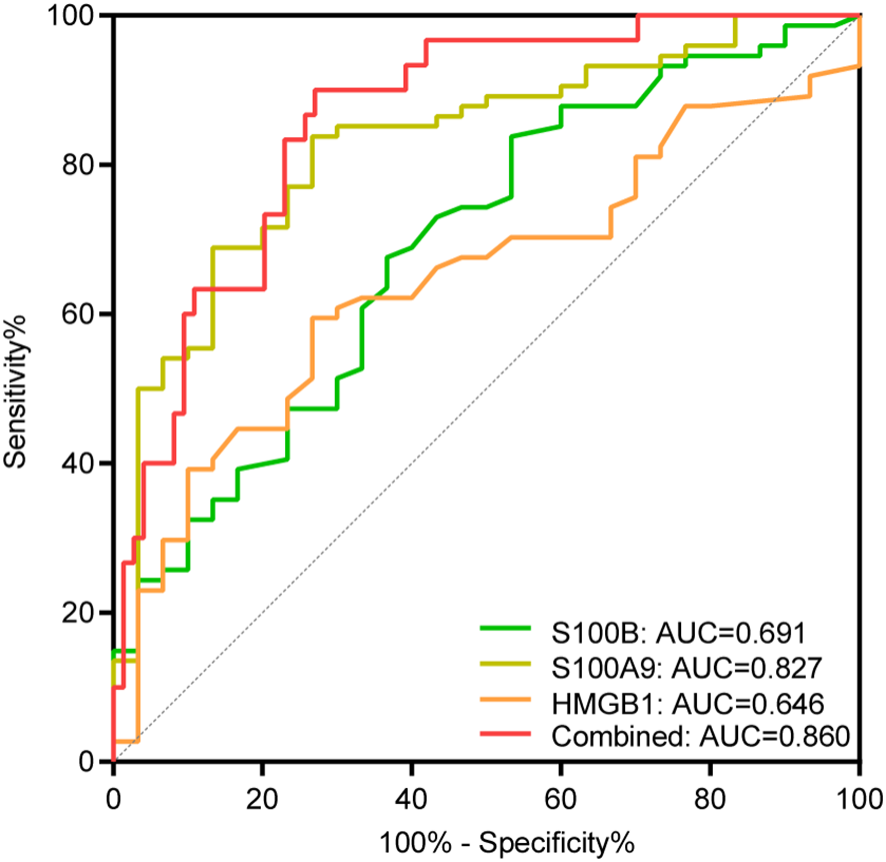
ROC curve analysis of S100B, S100A9, and HMGB1 for assessing disease activity. AUC indicates the area under the receiver operating characteristic curve. The green, yellow, and orange curve represents the ROC curve of S100B, S100A9 and HMGB1, respectively; the red curve represents the ROC curve of the combination of S100B, S100A9, and HMGB1.

We then found that the ROC curve analysis for assessing disease activity was significant for S100B, S100A9, and HMGB1, with AUC values of 0.691, 0.827, and 0.646, respectively. Importantly, the AUC value increased to 0.860 when the three alarmins mentioned above were combined, with a sensitivity of 90.00% and a specificity of 72.97% (**Figure 3**, **Table 4**). Although the diagnostic value of the combined model has a superimposed effect, the difference in actual values of S100A9 between the active and stable vitiligo patients is remarkable, indicating that the assessment of vitiligo activity based on the model with only one predictor is promising.

**Table 4:** Diagnostic values of biomarkers for the prediction of vitiligo activity.

Alarmins	Cut-off value	AUC (95%CI)	Sensitivity, %	Specificity, %	PPV, %	NPV, %
S100B, pg/ml	26.606	0.691 (0.580,0.802)	67.57	63.33	77.39	51.26
S100A9, ng/ml	917.498	0.827 (0.742,0.913)	83.78	73.33	85.37	70.88
HMGB1, ng/ml	1.476	0.646 (0.537,0.755)	59.46	73.33	80.55	49.34
S100B+S100A9		0.850 (0.772,0.929)	96.67	70.27	85.79	91.91
S100B+HMGB1		0.718 (0.613,0.824)	80.00	58.11	78.01	61.01
S100A9+HMGB1		0.848 (0.770,0.925)	86.67	75.68	86.87	75.35
all combined		0.860 (0.786,0.933)	90.00	72.97	86.08	79.71

AUC, area under the curve; PPV, positive predictive value; NPV, negative predictive value.

The diagnostic cut-off value corresponds to the maximum point of the Youden index.

The all combined represents the combination of S100B, S100A9, and HMGB1.

### Serum levels of S100B, S100A9, and HMGB1 correlate with the affected body surface area in patients with NSV

**Figure 4:**
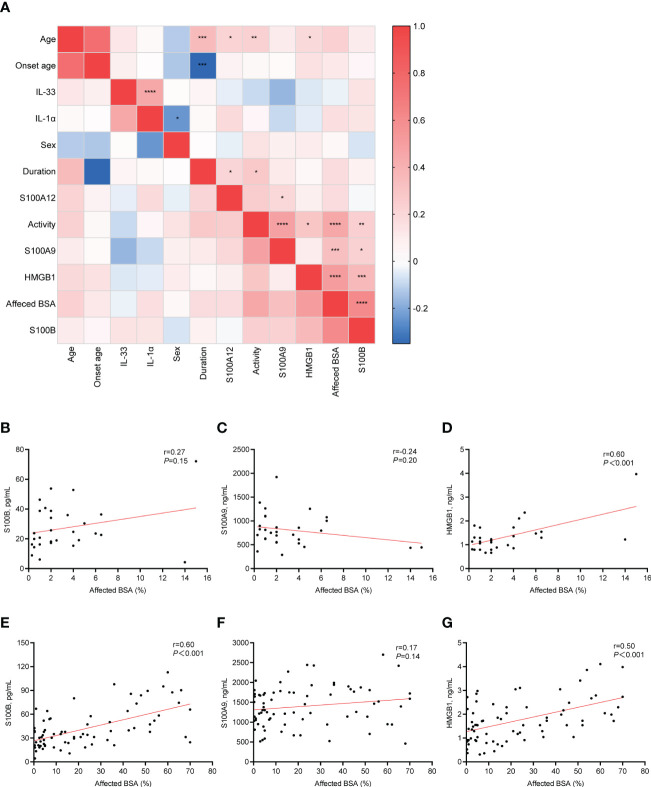
Correlation between clinical characteristics and serum alarmins. **(A)**, The correlation heatmap of clinical characteristics and serum alarmins by using Pearson correlations. **(B–D)**, Correlation changes in serum level of S100B, S100A9, and HMGB1 with changes in the affected BSA (%) of patients with stable vitiligo. **(E–G)**, Correlation changes in serum level of S100B, S100A9, and HMGB1 with changes in the affected BSA (%) of patients with active vitiligo. *P* values were calculated by Pearson correlation. *P < 0.05, **P < 0.01, ***P < 0.001, ****P < 0.0001.

We found that the serum levels of S100B, S100A9, and HMGB1 were positively correlated with increased BSA in NSV patients (r=0.61, *P <*0.001; r=0.33, *P <*0.001; r = 0.51, *P <*0.001, respectively) (**Figure 4A**). The heatmap also showed that the disease activity and severity of vitiligo were significantly correlated. Thus, we explored the association between S100B, S100A9, and HMGB1 and vitiligo severity in various disease activity phases (**Figures 4B–G**). The findings demonstrated that although S100B was only correlated with the affected BSA in active vitiligo (r =0.60, *P <*0.001) (**Figures 4B, E**), HMGB1 was associated with the vitiligo severity in both the stable and active stages (stable phase, r=0.60, *P <*0.001; active phase, r=0.50, *P <*0.001) (**Figures 4D, G**). However, no correlation was shown between affected BSA and S100A9 serum levels in patients with active and stable vitiligo (**Figures 4C, F**).

## Discussion

In the current study, we have evaluated six alarmins and demonstrated that S100B, S100A9, and HMGB1 as possible promising candidates for the biomarkers of NSV with high accuracy. In addition, the predictive accuracy for disease diagnosis and assessment was significantly improved when various alarmins were used in combination. This finding has special clinical and practical significance.

The clinical diagnosis of vitiligo is often determined by the appearance of reduced or lost skin pigmentation. However, this is not always the case. Vitiligo lesions at onset might be challenging to distinguish from adjacent normal skin due to the slight loss of pigmentation. Although histopathology is the gold standard for diagnosis and blister fluid testing could more accurately reflect the changes in the skin inflammatory microenvironment, these invasive examinations may induce the Koebner phenomenon in patients at the active stage. In contrast, circulating biomarkers are easier to collect and more acceptable for patients. Our ROC analysis revealed that serum IL-1α, S100B, S100A9, and HMGB1 had predictive capacity on the diagnosis of vitiligo. Of these, by using the cut-off value of serum S100B, approximately 92% of patients with NSV could be identified. Although these alarmins are also upregulated in other autoimmune diseases, we could infer that the overall diagnostic accuracy of vitiligo could be significantly improved by combining these biomarkers with the patients’ disease features and physical examination.

With the dual guarantee of multivariate logistic regression and ROC analysis, we found that serum HMGB1, S100B, and S100A9 can efficiently discriminate between patients with stable and active vitiligo, and can be used as potential biomarkers for the prediction of disease activity. It is now widely accepted that alarmins (such as HMGB1 and certain S100 proteins) can stimulate innate immunity, trigger antigen-presenting cells, and activate adaptive immune responses, playing a key role in the pathogenesis of inflammatory and autoimmune diseases (8, 20, 21). Our previous study has demonstrated that HMGB1 is released from melanocytes under oxidative stress, and then promotes the skin migration of CD8^+^ T cells and the maturation of dendritic cells, thus contributing to the formation of oxidative stress-induced autoimmunity in vitiligo (13). Therefore, the elevated serum levels of HMGB1 indicate a hyperactive immune state in patients with vitiligo. Consistent with our study, a recent study by Speeckaert et al. has also confirmed the elevated serum S100B levels in NSV patients at the active stage, and the *in vitro* experiments have shown that the release of S100B by stressed melanocytes may be the most plausible source of serum S100B in patients with NSV (14). Moreover, S100B inhibitor pentamidine could stop hair graying in the monobenzone-induced depigmentation mice model, suggesting S100B as a potential therapeutic target for vitiligo (14).

Importantly, our research revealed for the first time that circulating level of S100A9 was significantly elevated in NSV patients, and displayed high accuracy in disease activity assessment (AUC = 0.827), superior to sCD27 (AUC = 0.649) and sCD25 (AUC = 0.716) reported in the previous studies (6). More importantly, S100A9 is highly expressed and easily measured in serum samples, with expression levels hundreds of times higher than soluble CDs and chemokines such as CXCL10, thus might be expected to be one of the most valuable biomarkers of vitiligo activity (6, 22). A close correlation between elevated S100 proteins and disease activity has also been demonstrated in many inflammatory diseases, including rheumatoid arthritis, inflammatory bowel disease, as well as Alzheimer’s disease (23). It has been well accepted that S100A9 interacts with and actives Toll-like receptor-4 (TLR-4)-expressing cells *via* a specific binding site, thus promoting the pro-inflammatory signaling cascade (24). However, S100A9 usually exists as heterodimeric complexes with S100A8 and forms heterotetramers in the presence of calcium (25). Due to an autoinhibitory feedback mechanism induced by calcium-dependent tetramerization, the alarmins S100A8/S100A9 are just temporarily active in the local microenvironment (26). Therefore, restricting the activity of S100A8/S100A9 *via* blocking a specific TLR4-binding site is not only a specific anti-inflammatory therapy but also minimizing systemic side effects (26). Thus the exact function and intervention mechanism of S100A9 in the development of vitiligo is of great importance to be investigated.

Unlike our study, Li et al. (27) and Vaccaro et al. (16) demonstrated that serum levels of IL-33 were elevated in patients, and positively correlated with disease activity. The reason for the difference may be attributed to the baseline characteristics of participants, the sample size, as well as other confounding factors.

Identifying the factors that affect the severity of vitiligo is also crucial for the treatment of vitiligo. Our study showed that S100B was only correlated with affected BSA in active vitiligo, but HMGB1 was associated with the vitiligo severity in both the active and stable stages. It should be noted that we used the affected BSA to assess disease severity, which can be affected by inter- and intra-assessor variability. More accurate and reliable methods of assessing disease severity need to be investigated, to further validate the potential of candidate serum markers as indicators of disease severity.

In our study, we excluded the patients receiving immunosuppressive or NB-UVB therapy within 6 weeks before sample detection, thus to some extent excluding the potential effects of treatments on the serum level of alarmins. Importantly, increasing studies have suggested that alarmins could also be used as biomarkers to monitor the efficacy of treatment in several autoimmune diseases. In patients with rheumatoid arthritis, serum S100A8/A9 and IL-33 levels were reduced after infliximab or anti-TNF-α treatment, respectively (10, 28); and in Juvenile Idiopathic Arthritis patients, serum concentrations of S100A8/S100A9 decreased significantly after intraarticular triamcinolone therapy (29); serum HMGB1 levels were decreased after the treatment with statins or prednisolone in patients with granulomatous polyangiitis (30). The prognosis and recurrence of patients could also be predicted by serum alarmins. Serum levels of IL-33 have been found higher in eosinophilic granulomatosis patients with polyangiitis and Behçet disease patients with uveitis at relapse than that at the onset or during remission (31, 32). The recurrence of ANCA-associated vasculitis was also closely associated with the levels of S100A8/A9 (33). Therefore, future studies are needed to determine whether alarmins can serve as clinical bio-indicators for the prediction of treatment response, prognosis, and recurrence of vitiligo.

There are several limitations in our study. First, the cohort size was relatively small, and the single-center design may limit the generalizability of these results. Furthermore, the cross-sectional design and observational study did not allow causal inference. Additionally, our study focused mainly on the role of alarmins in the diagnosis and assessment of vitiligo and was unable to determine their potential contribution to the prognosis and prediction of treatment response in vitiligo. Moreover, our study only detected a few specific alarmins but did not screen all the alarmins. Above all, future studies based on multicenter and longitudinal samples are needed to explore the association between serum alarmins and clinical progression and prognosis.

## Conclusion

In conclusion, this study discovers the potential significance of serum IL-1α, S100B, S100A9, and HMGB1 in the auxiliary diagnosis of patients with NSV. Additionally, it is highlighted that serum S100B, S100A9, and HMGB1 might be potential biomarkers for assessing the activity and severity of NSV. Thereby, alarmins offer a versatile, noninvasive way for diagnosis and clinical condition monitoring of vitiligo.

## Data availability statement

The raw data supporting the conclusions of this article will be made available by the authors, without undue reservation.

## Ethics statement

The studies involving human participants were reviewed and approved by ethics committee of Xijing Hospital of the Fourth Military Medical University. The patients/participants provided their written informed consent to participate in this study. Written informed consent was obtained from the individual(s) for the publication of any potentially identifiable images or data included in this article.

## Author contributions

KH and WW have contributed equally to this work. WD and SW collected the blood samples. KH, WW, SL, and CL conceived and designed the experiments. KH, WW, and XW performed statistical analysis. KH and WW drafted the manuscript. SL and CL revised the manuscript. All authors contributed to the article and approved the submitted version.
